# Multi‐organ carcinogenicity by inhalation exposure to 2‐Bromopropane in rats

**DOI:** 10.1002/1348-9585.12388

**Published:** 2023-02-09

**Authors:** Hideki Senoh, Tatsuya Kasai, Shigeyuki Hirai, Yusuke Furukawa, Kyohei Misumi, Yuko Goto, Kenji Takanobu, Michiharu Matsumoto, Shoji Fukushima, Shigetoshi Aiso

**Affiliations:** ^1^ Japan Bioassay Research Center Japan Organization of Occupational Health and Safety Hadano Japan

**Keywords:** 2‐BP, 2‐bromopropane, carcinogenicity, inhalation, rat

## Abstract

**Objective:**

The purpose of this study was to investigate the carcinogenicity of 2‐bromopropane (2‐BP) in rats.

**Methods:**

Male and female F344 rats were exposed by whole body inhalation to 2‐BP vapor at concentrations of 0, 67, 200, and 600 ppm for 6 h/day, 5 days/week for 2 years.

**Results:**

All rats of both sexes exposed to 600 ppm died or became moribund within 85 weeks. Death/moribundity was caused by 2‐BP induced tumors. In males, significantly increased tumors were malignant Zymbal's gland tumors; sebaceous adenoma and basal cell carcinoma of the skin/appendage; adenocarcinoma of the small/large intestine; follicular cell adenoma of the thyroid; fibroma of the subcutis, and malignant lymphoma of the lymph node. In addition, an increased trend in tumor incidence was found in the preputial gland, lung, forestomach, pancreas islet, brain, and spleen. In females, significantly increased tumors were adenocarcinoma and fibroadenoma of the mammary gland, squamous cell papilloma of the vagina, and large granular lymphocytic leukemia of the spleen. In addition, an increased trend in tumor incidence was found in Zymbal's gland, the clitoral gland, skin, large intestine, pancreas islet, uterus, and subcutis. Particularly, malignant Zymbal's gland tumors were induced even in males exposed to the lowest concentration, 67 ppm.

**Conclusion:**

Two‐year inhalation exposure to 2‐BP resulted in multi‐organ carcinogenicity in rats. Based on sufficient evidence of carcinogenicity in this study, 2‐BP has the potential to be a human carcinogen.

## 1 INTRODUCTION

2‐Bromopropane (2‐BP, CAS No. 75–26‐3) is a colorless non‐flammable liquid. Annual production of 2‐BP in Japan was approximately 100 tons in 2019 (estimate),^1^ and release into the environment (atmosphere) in Japan was approximately 1.8 tons in fiscal year 2020.^2^ It is used as an intermediate in the synthesis of medicines, agricultural chemicals, and photosensitizers. 2BP is also a solvent with a high dermal absorption rate.^3^ Previously, chlorofluorocarbons (CFC) were used as solvents in open systems. However, CFC depletes the ozone layer, so 2‐BP is used as an alternative solvent. In a Korean electronic factory, male, and female workers exposed to solvent containing 2‐BP developed reproductive and hematopoietic disorders, such as ovarian failure, azoospermia, olizospermia, and anemia.^4^, ^5^, ^6^ Experimentally it has been demonstrated that in rats 2‐BP decreased spermatogenesis by adversely affecting spermatogonia followed by depletion of spermatocytes, spermatids, and spermatozoa, with subsequent testicular atrophy.^7^, ^8^ These studies also suggested that the atrophied testes may not regenerate completely. 2‐BP is also reported to act as a reproductive toxicant in rats and mice.^9^, ^10^

In one genotoxicity study, 2‐BP was positive in an Ames test using *S. typhimurium* TA100 in the presence of an activating system (S9+) and using TA1535 in the presence and absence of them S9 activating system (S9+, S9−).^11^ This study also reported that an in vitro chromosome aberration test using Chinese hamster lung (CHL) cells with 2‐BP showed negative results and an in vivo micronucleus test using rats bone marrow was also negative.^11^ JETOC also reports that 2‐BP induces mutagenicity in TA100 (S9−) and TA1535 (S9+, S9−).^12^ JETOC reports that the chromosome aberration test is positive using CHL cells for 6 h (S9+, S9−), 24 h (S9−) and 48 h (S9−).^13^ In addition, 2‐BP induced DNA damage, impaired functional antioxidant cellular defenses, and enhanced lipid peroxidation in primary cultured rat Leydig cells in vitro.^14^ When judging comprehensively, these data indicate that 2‐BP is genotoxic and especially mutagenic. Therefore, it is imperative that carcinogenicity studies of 2‐BP using experimental animals for health risk assessment of 2‐BP‐exposed workers be carried out.

## 2 MATERIALS AND METHODS

The present study was conducted with reference and compliance to the Organisation for the Economic Co‐operation and Development (OECD) Guideline for Testing of Chemicals 451,^15^ OECD Principles of Good Laboratory Practices (GLP)^16^ and “Standards to be Observed by Testing Institutions” of Ministry of Labour Japan.^17^ The rats were cared for in accordance with the Standards relating to the Care and Keeping and Reducing Pain of Laboratory Animals^18^ and was reviewed and approved by the Institutional Animal Care and Use Committee of the Japan Bioassay Research Center (JBRC).

### 2.1 Test substance

2‐BP of reagent grade (Wako 1st grade, greater than 99.7% pure) was obtained from Fujifilm Wako Pure Chemical Industries, LTD. Each lot of 2‐BP used in the present study was analyzed for identity by mass spectrometer and for stability by gas chromatography prior to and after use. The mass spectrometry peak was consistent with the literature values for 2‐BP, confirming the identity of 2‐BP. No aberrant gas chromatography peaks were detected before or after use of each lot substances, confirming the stability of the 2‐BP used in the present study. Additionally, 2‐BP in the chambers was monitored using gas chromatography, and no aberrant gas chromatographic peaks of 2‐BP were detected in the inhalation exposure chambers.

### 2.2 Animal and husbandry

F344/DuCrlCrlj (SPF) rats of male and female were obtained at 4 weeks of age from Charles River Japan, Inc. The rats were quarantined and acclimated for 2 weeks, and then divided by stratified randomization into 4 weight‐matched groups, The rats were housed individually in stainless‐steel wire hanging cages in stainless steel inhalation exposure chambers maintained at a temperature of 23 ± 2°C and at a relative humidity of 50 ± 20% with 12 times air changes/h. Fluorescent lighting was maintained at a 12‐h. light/dark cycle. All rats had free access to a γ‐irradiation‐sterilized commercial pellet diet (CR‐LPF, Oriental Yeast Co.) and sterilized water.

### 2.3 Experimental design

Groups of 50 rats of both sexes (6 week old at the commencement) were exposed by whole‐body inhalation to airflow containing 2‐BP vapor at target concentrations of 0 (a control), 67, 200, and 600 ppm for 6 h/day, 5 day/week for 104 weeks. The highest concentration of 600 ppm was based on the results of our previous 13‐week toxicity study (unpublished data) showing that inhalation exposure to 1000 ppm was non‐lethal, but body weight loss was severe, and lesions were noted in various organs (e.g., testis and hematopoietic system), and 300 ppm did not induce any life‐threatening disorders and body weight loss was slight. Therefore, exposure to 600 ppm was not expected to shorten the normal lifespan of the rats except as a result of tumorigenesis.

### 2.4 Exposure method of 2‐BP

Airflows containing 2‐BP at designated target concentrations were prepared using a vaporization technique. Compressed clean air was bubbled into the 2‐BP solution in a temperature‐controlled glass flask (22°C) to produce a saturated vapor mixture. The air/vapor mixture was then passed through a thermostatic condenser maintained at 17°C to cool and condense the 2‐BP. Finally, it was rewarmed to 22°C, and the air/vapor mixture was diluted with clean air, vaporized completely, and introduced into a spiral line mixer located above each chamber using flow meter. In this mixer, the 2‐BP air/vapor was diluted to the target concentration with clean air with controlled humidity and temperature, and supplied to the inhalation chamber. The concentration of 2‐BP in the chambers was monitored using gas chromatography every 15 min throughout the exposure period. The concentrations of 2‐BP for each dose group was 67.2 ± 0.3 (mean ± SD), 200.2 ± 0.6 and 600.9 ± 1.5 ppm: the 2‐BP in the inhalation chambers was maintained with high precision at a constant concentration.

### 2.5 Clinical and pathological evaluation of rats

All rats were observed daily for clinical signs and mortality. Rats were weighed and their food consumption was measured weekly for the first 14 weeks and every 4 weeks thereafter. All rats received a complete necropsy. At the time of terminal necropsy, all rats were euthanized under anesthesia, and the organs were removed, weighed, and examined. The tissues of all rats for microscopic examination were fixed in neutral buffered 10% formalin, and tissue sections were prepared and stained with H&E, and were examined microscopically.

### 2.6 Statistical analysis

Incidences of neoplastic lesions were statistically analyzed with Peto's test^19^ and Fisher's exact test. Incidences of non‐neoplastic lesions were analyzed by chi‐square test with severity. Body weight, food consumption, and organ weight were analyzed by Dunnett's test. The statistical tests are described in detail in a previous paper.^20^ Survival curves were plotted according to the method of Kaplan and Meier,^21^ and the log‐rank test^22^ was used to test the statistical significance of the difference in survival rate between 2‐BP‐exposed groups and the controls.

## 3 RESULTS

### 3.1 Survival, body weight, and food consumption

**FIGURE 1:**
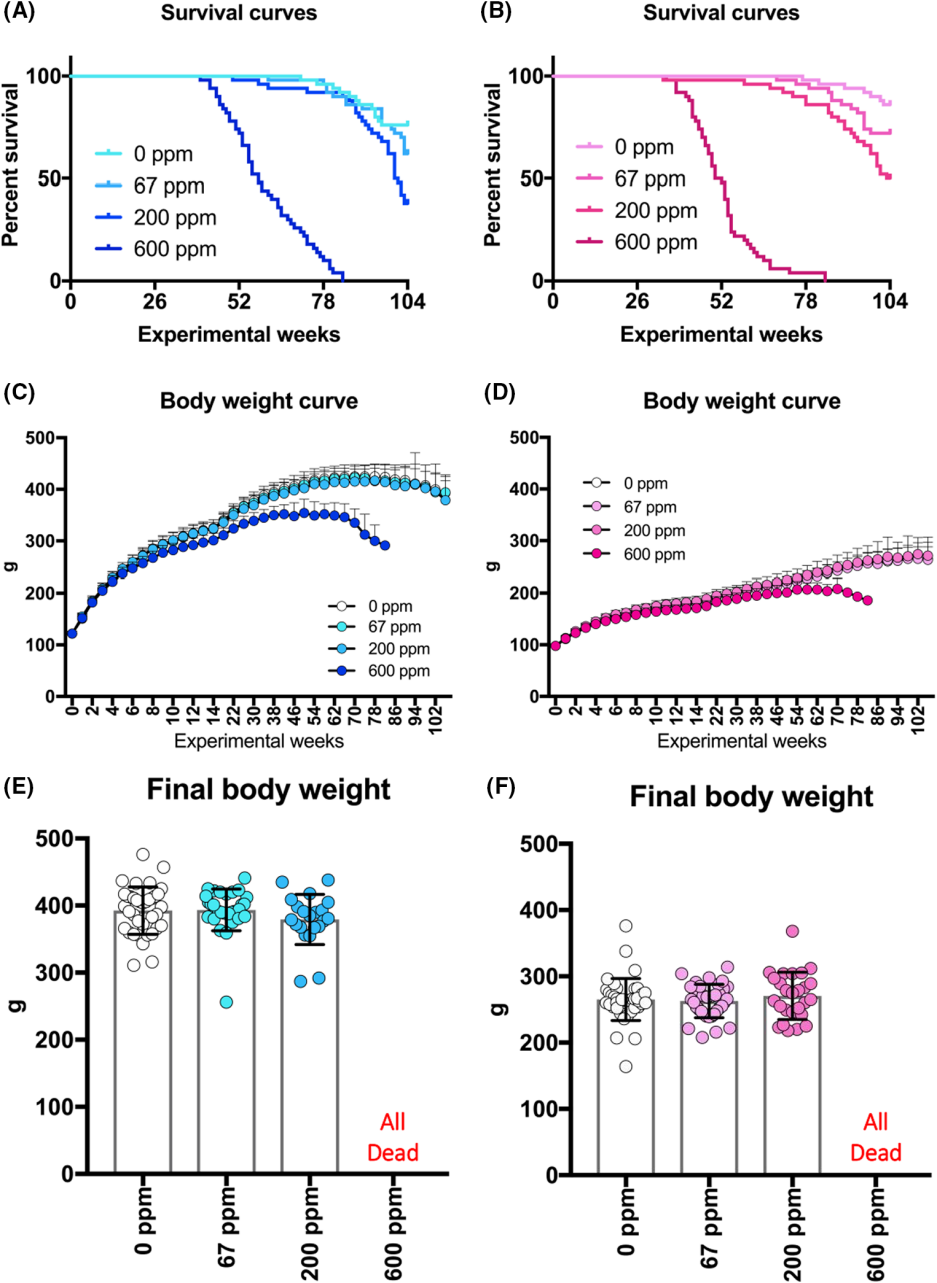
Survival curves, body weight curves, and final body weights. (A) Survival curves of male. (B) Survival curves of female. (C) Body weight curves of male. (D) Body weight curves of female. (E) Final body weight of male. (F) Final body weight of female.

All rats in the 600 ppm inhalation groups, both male, and female, died or moribund within week 85. Survival rates were also decreased in the 67 and 200 ppm inhalation groups, both male and female, compared to their respective controls by log‐rank test analysis (Figure 1A,B). In the 600 ppm inhaled group, the onset of tumor‐related deaths or moribundity was observed in males at week 41 and females at week 36, and survival rates fell below 50% at week 59 for males and week 53 for females. Most of the deaths were due to tumors: the tumors were Zymbal's gland tumors and skin/appendix tumors in males and mammary gland tumors in females. The terminal survival rates at the end of the 2‐year exposure period in males were control 76%, 67 ppm 62%, 200 ppm 38%, and 600 ppm 0%. In females, the survival rates at the end of the 2‐year exposure period were control 86%, 67 ppm 72%, 200 ppm 50%, and 600 ppm 0%.

In the 600 ppm group, suppression of body weight was observed from week 4 in males and from week 5 in females, and suppression of body weight by 10% or more was observed from week 30 in males and week 62 in females (Figure 1C,D). In contrast, in the 67 and 200 ppm groups of males and females, there was no statistical difference from the control group in body weight throughout the study. The final body weight at week 104 was 99% or more of the control group for both males and females (Figure 1E,F).

In the 600 ppm male and female groups, food consumption was lower during the latter half of the exposure period compared to their respective control groups. In contrast, in the 67 and 200 ppm male and female groups, food consumption was slightly higher than the controls at the beginning of exposure and was the same as the controls thereafter (data not shown).

### 3.2 Pathology

#### 3.2.1 Organ weight

Some organs showed significant differences in absolute weight of the 200 ppm male and female groups (male: low value for the brain; female: low value in the brain and high values for the kidney and spleen), but there were no differences in the relative organ weights of either the male or female 200 ppm groups at the end of the study (data not shown).

#### 3.2.2 Tumors in male rats

##### Zymbal's gland

**FIGURE 2:**
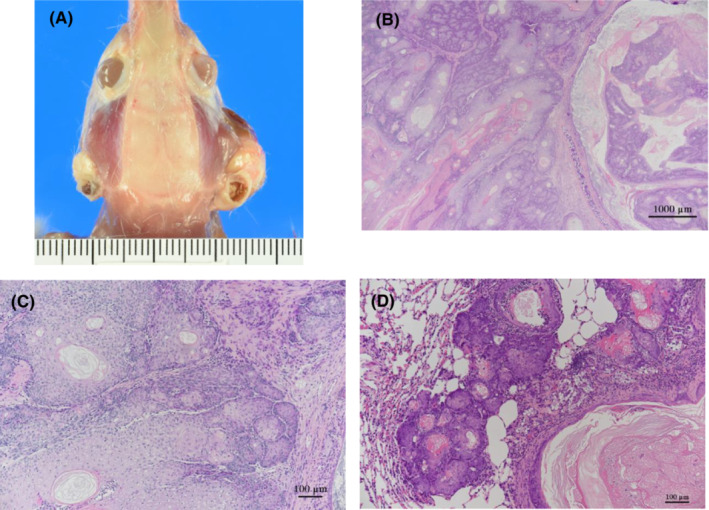
Zymbal's gland tumor. (A) A macroscopic image of the canal nodules (bilateral) of a male rat with malignant Zymbal's gland tumors. Malignant Zymbal's gland tumors are observed on both sides (male, 600 ppm group). (B) Malignant Zymbal's gland tumor (male, 600 ppm group). The tumor is characterized by the papillary structures of epithelial cells composed of a both of squamous cells and sebaceous cells. (C) High magnification of B. (D) Lung metastasis of malignant Zymbal's gland tumor (male, 600 ppm group).

Malignant Zymbal's gland tumors were found in 0/50, 5/50, 6/50, and 23/50 rats in the 0, 67, 200, and 600 ppm groups, respectively, and were significantly increased in all exposure groups by the Fisher extract test and showed a positive trend by Peto's test (Table 1). The combined incidence of benign and malignant Zymbal's gland tumors were also significantly increased in all exposure groups by the Fisher extract test and showed a positive trend by Peto's test. Zymbal's gland tumors were sometimes bilateral, and malignant Zymbal's gland tumors showed cranial‐destroying growth and were often the cause of death or moribundity, and rarely metastasized to the lung (Figure 2).

**TABLE 1:** Number of tumor‐bearing F344 rats exposed to 2‐BP for 2 years and historical control data in male.

	Exposed to 2‐BP									Historical control data^a^					
Group (ppm)	0		67		200		600		Peto‘s test	Animals with tumors	Rate^b^		Range		
Number of animals	50		50		50		50				%		%		
Zymbal gland															
Zymbal gland tumor: benign (A)	0		0		1		2			14	0.4		0	―	4
Zymbal gland tumor: malignant (B)	0		5*		6*		23**		↑↑	21	0.6		0	―	4
A and/or B	0		5*		7**		25**		↑↑	―	―			―	
Skin/appendage															
Squamous cell papilloma (C)	1		0		1		2			42	1.2		0	―	8
Basal cell epithelioma (D)	0		0		2		3		↑↑	2	0.1		0	―	2
Keratoacanthoma (E)	4		5		7		6		↑↑	125	3.5		0	―	14
Sebaceous adenoma (F)	0		1		2		10**		↑↑	15	0.4		0	―	4
Squamous cell carcinoma (G)	0		1		0		1			11	0.3		0	―	4
Basal cell carcinoma (H)	0		0		0		12**		↑↑	3	0.1		0	―‐	2
C, D, E, F, G, and/or H	5		6		9		22**		↑↑	―	―			―	
Preputial gland															
Adenoma (I)	0		0		1		4		↑↑	92	2.6		0	―	12
Adenocarcinoma (J)	0		1		0		0			4	0.1		0	―	4
I and/or J	0		1		1		4		↑↑	―	―			―	
Lung															
Bronchiolo‐alveolar adenoma (K)	3		7		5		7		↑↑	143	4.0		0	―	12
Bronchiolo‐alveolar carcinoma (L)	1		3		1		2			31	0.9		0	―	8
Squamous cell carcinoma (M)	0		0		0		1			2	0.1		0	―	2
K, L, and/or M	4		8		6		9		↑↑	―	―			―	
Forestomach															
Squamous cell papilloma (N)	0		0		1		4		↑↑	6	0.2		0	―	2
Squamous cell carcinoma (O)	0		0		0		1			7	0.2		0	―	2
N and/or O	0		0		1		5*		↑↑	―	―			―	
Small intestine															
Adenocarcinoma	0		0		2		7**		↑↑	1	0.0		0	―	2
Large intestine															
Adenoma (P)	0		0		1		3		↑↑	1	0.0		0	―	2
Adenocarcinoma (Q)	0		1		6*		8**		↑↑	0	0		0	―	0
P and/or Q	0		1		7**		11**		↑↑	―	―			―	
Pancreas islet															
Islet cell adenoma (R)	3		2		5		1		↑	252	7.1		0	―	14
Islet cell carcinoma (S)	0		1		2		0			24	0.7		0	‐	4
R and/or S	3		3		7		1		↑↑	―	―			―	
Thyroid															
Follicular adenoma (T)	0		1		5*		2		↑↑	34	1.0		0	―	4
Follicular adenocarcinoma (U)	0		3		1		0			41	1.2		0	―	8
T and/or U	0		4		6*		2		↑↑	―	―			―	
Subcutis															
Hemangioma	0		0		3		1		↑↑	4	0.1		0	―	2
Fibroma (V)	7		5		15*		5		↑↑	307	8.7		2	―	20
Fibrosarcoma (W)	0		0		1		0			14	0.4		0	―	4
V and/or W	7		5		16*		5		↑↑	―	―			―	
Brain															
Astrocytoma^c^	0		2		4		2		↑↑	26	0.7		0	―	4
Lymph node															
Malignant lymphoma	1		0		3		7*		↑↑	6	0.2		0	―	2
Spleen															
LGL leukemia^d^	10		7		16		4		↑↑	418	11.8		2	―	32

*Note*: * and **: Significantly different at *p* ≦ .05 and *p* ≦ .01 by Fisher's exact test, respectively. ↑ and ↑↑: Significantly different at *P* ≦ .05 and *P* ≦ .01 by Peto's test, respectively.

^a^ Historical control data since 1987 (71 studies, 3548 rats).

^b^ Percentage of animals with tumors.

^c^ Historical control data is glioma (astrocytoma, mixed glioma and/or oligodendroglioma).

^d^ Large granular lymphocytic leukemia.

##### Skin/appendage

**FIGURE 3:**
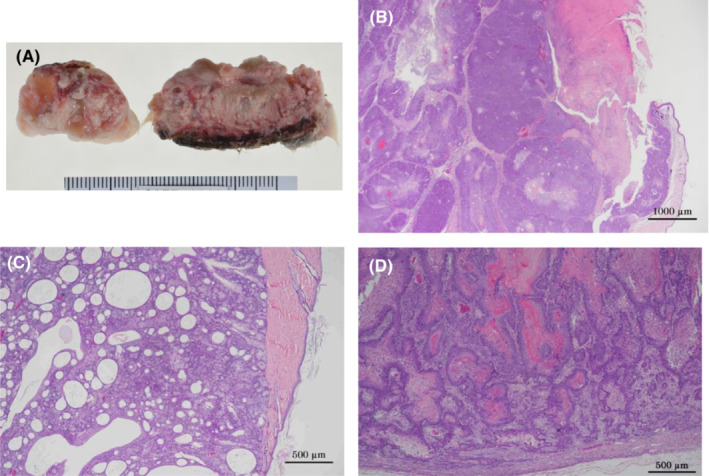
Skin/appendage tumor. (A) Macroscopic image of skin/appendage nodules, (B) Basal cell carcinoma (male, 600 ppm group). (C) Sebaceous adenoma of the skin/appendage (male, 600 ppm group), (D) Squamous cell carcinoma of the Skin (male, 600 ppm group).

There was a positive trend by Peto's test in the incidence of sebaceous adenomas and basal cell carcinomas, and the incidences were significantly increased in the 600 ppm group by the Fisher extract test. There was also a positive trend by Peto's test in the incidences of basal cell epithelioma and keratoacanthoma. In addition, there was a positive trend by Peto's test in the combined incidence of skin/appendage tumors (squamous cell papilloma, basal cell epithelioma, keratoacanthoma, sebaceous adenoma, squamous cell carcinoma, and basal cell carcinoma), and the combined incidence was significantly increased in the 600 ppm group by the Fisher extract test. Multiple skin/appendage tumors sometimes developed in a single rat (Figure 3).

##### Preputial gland

There was a positive trend by Peto's test in the incidence of adenomas and in the combined incidence of adenomas and adenocarcinomas in the preputial gland.

##### Lung

There was a positive trend by Peto's test in the incidence of bronchiolo‐alveolar adenomas and in the combined incidence of bronchiolo‐alveolar adenomas, bronchiolo‐alveolar carcinomas, and squamous cell carcinomas in the lung.

##### Gastrointestinal tract

**FIGURE 4:**
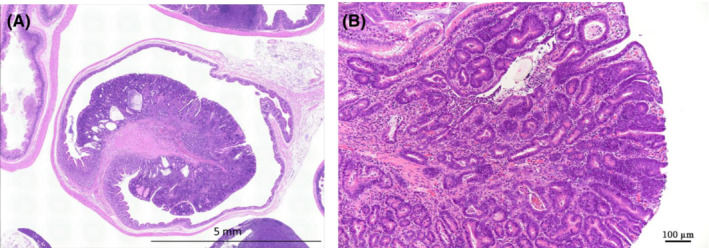
Large intestine tumor. (A) Adenocarcinoma of the large intestine (male, 600 ppm group). (B) High magnification of A. The tumor has structural atypia and cellular atypia.

In the forestomach, there was a positive trend by Peto's test in the incidence of squamous cell papillomas and in the combined incidence of squamous cell papillomas and squamous cell carcinomas, and the combined incidence of squamous cell papillomas and squamous cell carcinomas was significantly increased in the 600 ppm group by the Fisher extract test. In the small intestine, there was a positive trend by Peto's test in the incidence of adenocarcinomas, and the incidence was significantly increased in the 600 ppm group by the Fisher extract test. In the large intestine, there was a positive trend by Peto's test in the incidence of adenomas, adenocarcinomas, and the combined incidence of adenomas and adenocarcinomas, and the incidence of adenocarcinomas and the combined incidence of adenomas and adenocarcinomas was significantly increased in the 200 ppm and 600 ppm groups by the Fisher extract test. Some of the small intestine and large intestine adenocarcinomas showed moderate to severe atypia and invasive growth into the muscular layer (Figure 4).

##### Endocrine system

In pancreas islet, there was a positive trend by Peto's test in the incidence of islet cell adenomas and in the combined incidence of islet cell adenomas and islet cell carcinomas. In the thyroid, there was a positive trend by Peto's test in the incidence of follicular adenomas and the combined incidence of follicular adenomas and follicular adenocarcinomas, and the incidence of follicular adenomas and the combined incidence of follicular adenomas and follicular adenocarcinomas were significantly increased in the 200 ppm group by the Fisher extract test.

##### Subcutis

There was a positive trend by Peto's test in the incidence of fibromas and combined incidence of fibromas and fibrosarcomas, and the incidence of fibromas and the combined incidence of fibromas and fibrosarcomas were significantly increased in the 200 ppm group by the Fisher extract test. There was also a positive trend by Peto's test in the incidence of hemangiomas.

##### Brain

There was a positive trend by Peto's test in the incidence of astrocytomas.

##### Hematopoietic system

There was a positive trend by Peto's test in the incidence of malignant lymphomas of the lymph node, and the incidence was significantly increased in 600 ppm groups by the Fisher extract test. There was also a positive trend by Peto's test in the incidence of large granular lymphocytic (LGL) leukemia of the spleen.

#### 3.2.3 Non‐neoplastic lesion in male rats

Epithelial hyperplastic lesions were increased in the lung and tongue. Bronchiolo‐alveolar hyperplasia of the lung and squamous cell hyperplasia of the tongue were significantly increased in 600 ppm groups by chi‐square test. There were no epithelial hyperplastic lesions in other organs, including the intestinal tract. In the testis, seminiferous tubular atrophy was significantly increased in 600 ppm groups by chi‐square test, and most of the rats in the 600 ppm group had severe seminiferous tubular atrophy (no spermatogenesis).

#### 3.2.4 Tumors in female rats

##### Mammary gland

**FIGURE 5:**
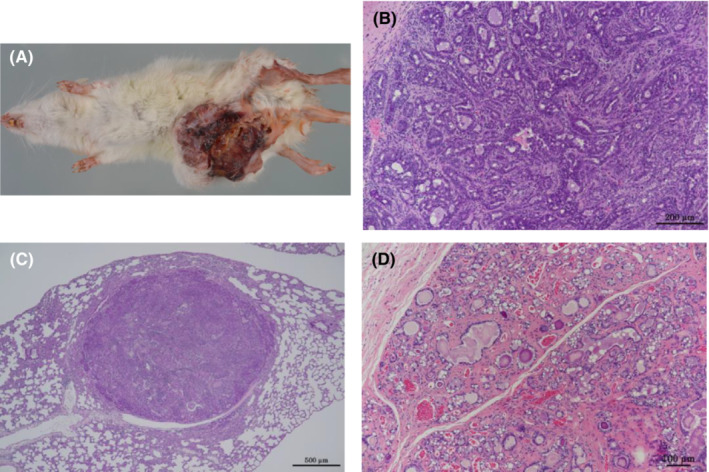
Mammary gland tumor. (A) A macroscopic image of a mammary gland tumor (female, 600 ppm group). (B) Adenocarcinoma of the mammary gland (female, 600 ppm group). (C) Metastasis of mammary gland adenocarcinoma to the lung, (female, 600 ppm group). (D) Fibroadenoma of the mammary gland (female, 200 ppm group).

Adenocarcinomas were found in 0/50, 2/50, 5/50, and 48/50 female rats in the 0, 67, 200, and 600 ppm groups, respectively (Table 2). The adenocarcinoma incidence showed a positive trend by Peto's test and was significantly increased in the 200 ppm and 600 ppm groups by Fisher extract test. There was also a positive trend by Peto's test in the incidence of fibroadenomas, adenomas, and the combined incidence of adenomas, fibroadenomas, adenocarcinomas, and adenosquamous carcinomas. The incidence of fibroadenomas was significantly increased in the 200 ppm by Fisher extract test and the combined incidence of adenomas, fibroadenomas, adenocarcinomas, and adenosquamous carcinomas was significantly increased in the 200 ppm and 600 ppm groups by Fisher extract test. Multiple mammary tumors sometimes developed in a single rat. The mammary adenocarcinomas often showed hemorrhage, and were often the cause of death or moribundity. Adenocarcinomas rarely metastasized to the lung (Figure 5).

**TABLE 2:** Number of tumor‐bearing F344 rats exposed to 2‐BP for 2 years and historical control data in female.

	Exposed to 2‐BP									Historical control data^a^					
Group (ppm)	0		67		200		600		Peto‘s test	Animals with tumors	Rate^b^		Range		
Number of animals	50		50		50		50				%		%		
Mammary gland															
Adenoma (A)	1		0		5		0		↑↑	65	1.9		0	―	18
Fibroadenoma (B)	2		4		13**		1		↑↑	391	11.7		0	―	28
Adenocarcinoma (C)	0		2		5*		48**		↑↑	36	1.1		0	―	6
Adenosquamous carcinoma (D)	0		0		1		0			0	0		0	―	0
A, B, C and/or D	3		6		21**		48**		↑↑	―	―			―	
Zymbal gland															
Zymbal gland tumor: benign (E)	0		0		0		1			9	0.3		0	―	4
Zymbal gland tumor: malignant (F)	0		1		1		4		↑↑	13	0.4		0	‐	6
E and/or F	0		1		1		4		↑↑	―	―			―	
Clitoral gland															
Adenoma (G)	1		1		4		4		↑	100	3.0		0	―	10
Squamous cell papilloma (H)	0		0		0		1			3	0.1		0	‐	2
Adenocarcinoma (I)	0		0		1		1			0	0		0	―	0
G, H and/or I	1		1		5		6		↑↑	―	―			―	
Skin/ appendage															
Squamous cell papilloma (J)	2		0		0		4		↑↑	10	0.3		0	―	4
Trichoepithelioma (K)	1		0		0		0			8	0.2		0	―	2
Basal cell epithelioma (L)	0		0		0		1			0	0		0	―	0
Keratoacanthoma (M)	1		0		0		0			11	0.3		0	―	4
Squamous cell carcinoma (N)	0		0		1		0			6	0.2		0	―	2
J, K, L, M and/or N	4		0		1		5		↑↑	―	―			―	
Large intestine															
Adenoma (O)	0		0		2		2			0	0		0	―	0
Adenocarcinoma (P)	0		0		0		2			1	0		0	―	2
O and/or P	0		0		2		4		↑↑	―	―			―	
Pancreas islet															
Islet cell adenoma (Q)	1		0		4		0		↑	37	1.1		0	―	4
Islet cell carcinoma (R)	0		1		0		0			6	0.2		0	‐	2
Q and/or R	1		1		4		0		↑	―	―			―	
Uterus															
Adenoma (S)	1		0		0		2			9	0.3		0	―	2
Adenocarcinoma (T)	1		0		1		2			29	0.9		0	―	6
S and/or T	2		0		1		4		↑↑	―	―			―	
Endometrial stromal polyp	9		4		11		8		↑	487	14.6		2	―	28
Vagina															
Squamous cell papilloma (U)	1		2		7*		4		↑↑	3	0.1		0	―	2
Squamous cell carcinoma (V)	0		0		1		0			1	0.0		0	―	2
U and/or V	1		2		8*		4		↑↑	―	―			―	
Subcutis															
Fibroma (W)	2		1		4		0		↑	36	1.1		0	―	8
Fibrosarcoma (X)	0		0		1		2			5	0.1		0	―	2
W and/or X	2		1		5		2		↑↑	―	―			―	
Spleen															
LGL leukemia^c^	2		6		10*		1		↑↑	406	12.1		2	―	26

*Note*: * and **: Significantly different at *P* ≦ .05 and *P* ≦ .01 by Fisher’s exact test, respectively. ↑ and ↑↑: Significantly different at *P* ≦ .05 and *P* ≦ .01 by Peto's test, respectively.

^a^ Historical control data since 1987 (67 studies, 3347 rats).

^b^ Percentage of animals with tumors.

^c^ Large granular lymphocytic leukemia.

##### Zymbal's gland

There was a positive trend by Peto's test in the incidence of malignant Zymbal's gland tumors and the combined incidence of benign and malignant Zymbal's gland tumors.

##### Clitoral gland

There was a positive trend by Peto's test in the incidence of adenomas and the combined incidence of adenomas, squamous cell papillomas, and adenocarcinomas.

##### Skin/appendage

There was a positive trend by Peto's test in the incidence of squamous cell papillomas and the combined incidence of squamous cell papillomas, trichoepitheliomas, basal cell epitheliomas, keratoacanthomas, and squamous cell carcinomas.

##### Gastrointestinal tract

In large intestine, there was a positive trend by Peto's test in the combined incidence of adenomas and adenocarcinomas.

##### Pancreas islet

There was a positive trend by Peto's test in the incidence of islet cell adenomas and the combined incidence of islet cell adenomas and islet cell carcinomas.

##### Reproductive system

In the uterus, there was a positive trend by Peto's test in the combined incidence of adenomas and adenocarcinomas, and endometrial stromal polyps also showed a positive trend by Peto's test. In the vagina, there was a positive trend by Peto's test in the incidence of squamous cell papillomas and the combined incidence of squamous cell papillomas and squamous cell carcinomas, and their incidences were significantly increased in the 200 ppm group by Fisher extract test.

##### Subcutis

There was a positive trend by Peto's test in the incidence of fibromas and the combined incidence of fibromas and fibrosarcomas.

##### Hematopoietic system

There was a positive trend by Peto's test in the incidence of LGL leukemias of the spleen, and the incidence was significantly increased in the 200 ppm group by the Fisher exact test.

#### 3.2.5 Non‐neoplastic lesions in female rats

Epithelial hyperplastic lesions were found in the vagina, and were significantly increased in 200 ppm group by chi‐square test. There were no histopathological findings related to 2‐BP exposure in the ovary or uterus in any of the groups.

## 4 DISCUSSION

In this OECD guideline and GLP‐compliant carcinogenicity study, 2‐year inhalation exposure to 2‐BP at concentrations of 67, 200, and 600 ppm of male and female F344 rats significantly induced malignant and/or benign tumors in multiple organs.

Inhalation exposure to 2‐BP affected the survivals of male and female rats. All rats in the 600 ppm groups, both male, and female, died/moribund by week 85, and terminal survival rates were decreased in the 67 ppm and above groups. The cause of death was 2‐BP induced tumors. Average body weight was significantly suppressed in both male and female 600 ppm groups. Food consumption was also lower in both male and female in the 600 ppm group. Body weight and food consumption in the 67 and 200 ppm groups were not different from the control groups.

In this study, tumors developed in multiple organs in male and female rats. In males, significantly increased tumor incidence was found in the Zymbal's gland, skin/appendage, forestomach, small intestine, large intestine, thyroid, subcutis, and lymph node, and an increased trend in tumor incidence was found in the preputial gland, lung, pancreas islet, brain, and spleen. In females, significantly increased tumor incidence was found in the mammary gland, vagina, and spleen, and an increased trend in tumor incidence was found in Zymbal's gland, clitoral gland, skin, large intestine, pancreas islet, uterus, and subcutis.

In particular, tumor developments in the Zymbal's gland, skin/appendage, forestomach, small intestine, and large intestine were more prominent in males than in females. In addition, multiple tumors developed in the study were Zymbal's gland and skin/appendage tumors in males and mammary gland tumors in females. These tumors developed early, and were the most common tumors. Notably, Zymbal's gland tumors in males were significantly increased even at the lowest concentration, 67 ppm. In the females, mammary gland tumors developed in 200 ppm and above. Overall, induction of tumors including malignancy occurred in multiple organs and sex differences were observed.

The concentrations of 2‐BP used in this study were decided based on the results of a previous 13‐week study. In that study, exposure to 300 ppm 2‐BP did not cause significant body weight loss. In the present study, there was little suppression of body weight in any of the exposed groups during the initial 13 weeks of the study. Suppression of body weight by 10% or more was observed from week 30 in males and week 62 in females in the 600 ppm‐exposed groups, and the onset of tumor death in the 600 ppm groups was 41 weeks for males and 36 weeks for females. Thus, the suppression of body weight in the 600 ppm groups was due to tumor development, and not to exposure to 2‐BP concentrations above maximum tolerated dose. In addition, it is important that there was significant induction of tumors development in the 67 ppm and 200 ppm male groups and the 200 ppm female group despite the fact that body weights in these groups were the same as the control groups.

The multi‐organ carcinogenicity and sex differences in tumor development induced by 2‐BP in this study suggest that in different organs, 2‐BP exert different DNA reactivities that are involved in the initiation stage of multistage carcinogenesis. This is consistent with a genotoxic agent. Thus, this study supports the premise that 2‐BP is genotoxic and able to induce DNA mutations in multiple organs. However, further study on the mechanism of multiorgan carcinogenicity mediated by 2‐BP is required. Moreover, mechanism of sex difference is also required.

A characteristic feature of 2‐BP‐induced tumors was the development of intestinal tumors, especially large intestinal tumors. These tumors are extremely rare in rats. The spontaneous tumor incidences of adenomas/carcinomas in the small intestine/large intestine at JBRC are under 0.03% in both males and females (unpublished data). In addition, the fact that 2‐BP showed large intestine carcinogenicity is a very important finding for comparison with humans.

There are no equivalent organ/tissue to the Zymbal's gland and forestomach in humans, and this must be taken into consideration when extrapolating Zymbal's gland and forestomach tumorigenesis in rats to humans for cancer risk assessment. However, in the case of carcinogenicity due to the genotoxic substances the biological properties of the genotoxic carcinogens are applicable to humans. Consequently, the carcinogenicity of a genotoxic agent can be extrapolated to humans for risk assessment. In addition, the as low as reasonably achievable (ALARA) approach is widely applicable to genotoxic carcinogens. Therefore, Zymbal's gland, and forestomach carcinogenicities, especially that of Zymbal's gland, in males exposed to 2‐BP is a very important result.

Generally, genotoxic and multiorgan‐targeting carcinogens such as *N*‐nitroso compounds and heterocyclic amines seem to be human carcinogens.^23^ 2‐BP was also found to be a multi‐organ carcinogen in this study, supporting the premise that 2‐BP is possibly a human carcinogen.

Carcinogens are classified into genotoxic and non‐genotoxic types. For quantitative risk assessment of carcinogens, the theory that genotoxic carcinogens do not have a threshold dose is applied (non‐threshold theory). However, this is not realistic from the viewpoint of occupational health. Therefore, to estimate the quantitative relationship between exposure concentration and 2‐BP carcinogenicity, a benchmark dose tool is used to obtain a point of departure for risk assessment. BMDL_10_ for tumor development in the Zymbal's gland in this study is 47 ppm using EPA software.^24^ This BMDL_10_ will be important for setting exposure limit and calculating the margin of exposure in the workplace for the carcinogenicity of 2‐BP, and it will contribute to the cancer risk assessment to humans of 2‐BP.

JBRC performed a 26‐week inhalation exposure study of 2‐BP using the rasH2 mouse model (unpublished data). Male and female mice were exposed to 0, 67, 200, or 600 ppm 2‐BP for 6 h/day, 5 days/week for 26 weeks. There was a positive trend by Peto's test in the incidence of lung tumor development in male and female mice.

1‐Bromopropane (1‐BP) was tested for inhalation carcinogenicity of rats and mice in the National Toxicology Program (NTP).^25^ NTP concluded that 1‐BP exerted carcinogenicity in many organs of rats and mice including the skin in male rats, large intestine in male and female rats, and the lung in female mice. The NTP conducted a cancer evaluation on 1‐BP for possible listing in the Report on Carcinogens (RoC)^26^ using its own studies and assessments based on the available scientific evidence of 1‐BP carcinogenicity, and the NTP recommended that 1‐BP be listed as reasonably anticipated to be a human carcinogen in the RoC. Notably, tumors induced by 2‐BP and 1‐BP were observed mostly the same organs and the tumor types were remarkably similar. However, this 2‐BP carcinogenicity study in rats showed more multiorgan carcinogenicity including induction of non‐epithelial tumors.

## 5 CONCLUSION

Two year inhalation exposure to rats of 2‐BP exerted multi‐organ carcinogenicity in the present study, resulting in sufficient evidence for the carcinogenicity. Furthermore, it is considered that 2‐BP has the potential to be a human carcinogen based on the results of the present study and above points of view.

## AUTHOR CONTRIBUTIONS

Hideki Senoh conducted histopathology and drafted the manuscript. Tatsuya Kasai and Shigeyuki Hirai conducted administration and analysis of test substance. Kyohei Misumi conducted animal care take. Kenji Takanobu conducted histopathology. Yuko Goto and Yusuke Furukawa drafted the manuscript of administration and chemical analysis of test substance, and reviewed the results. Michiharu Matsumoto conducted statistical analysis and reviewed results. Shoji Fukushima and Shigetoshi Aiso supervised study and reviewed manuscript. All the authors read and approved the final manuscript.

## DISCLOSURE

*Approval of the research protocol*: The present study was conducted with reference and compliance to the Organisation for the Economic Co‐operation and Development (OECD) Guideline for Testing of Chemicals 451, OECD Principles of Good Laboratory Practices (GLP) and “Standards to be Observed by Testing Institutions” of Ministry of Labour Japan. *Informed Consent*: N/A. *Registry and the Registration No. of the study*: Study number of the Japan Bioassay Research Center (JBRC) was 0877. *Animal Studies*: The rats were cared for in accordance with the Standards relating to the Care and Keeping and Reducing Pain of Laboratory Animals and was reviewed and approved by the Institutional Animal Care and Use Committee of the Japan Bioassay Research Center (JBRC). Approval number was 0144. *Conflict of interest statement*: The authors declare no conflict of interest.

## Data Availability

The data that support the findings of this study are openly available in “Syokuba no anzen site” in Ministry of Health, Labour and Welfare (https://www.mhlw.go.jp/) at https://anzeninfo.mhlw.go.jp/user/anzen/kag/pdf/gan/0877TABLE.pdf, https://anzeninfo.mhlw.go.jp/user/anzen/kag/pdf/gan/0877FIGURE.pdf.
